# Revisiting the mechanisms linking blood glucose to cognitive impairment: new evidence for the potential important role of klotho

**DOI:** 10.3389/fendo.2024.1323407

**Published:** 2024-03-05

**Authors:** Xiangliang Liu, Yuguang Li, Xinqiao Chen, Hongmei Yin, Fangqi Li, Naifei Chen, Jiuwei Cui, Wei Li

**Affiliations:** ^1^ Cancer Center, The First Affiliated Hospital of Jilin University, Changchun, China; ^2^ Department of General Practice, The First Affiliated Hospital of Jilin University, Changchun, China

**Keywords:** glucose, klotho, cognitive function, cognitive impairment, hyperglycemia

## Abstract

**Background:**

The association between blood glucose and cognition is controversial. Klotho is an anti-aging protein with neural protective effects. This study aimed to use a population-based study to disentangle the relationship between blood glucose levels and cognitive function in older adults, and to explore the role of klotho in it.

**Methods:**

A total of 1445 eligible participants from National Health and Nutrition Examination Survey (NHANES) 2011-2014 were included in our study. Cognitive function was assessed by Digit Symbol Substitution Test (DSST) and categorized into four quartiles (Q1-Q4). General characteristics and laboratory test results including serum klotho concentration and blood glucose levels were collected. Associations of cognitive function and klotho levels with blood glucose concentrations were explored through multivariate linear regression models. Mediation models were constructed to figure out the mediating role of klotho.

**Results:**

All three multivariate linear regression models showed a negative correlation between blood glucose and cognitive function. (Model 1, β=-0.149, 95%CI: -0.202,-0.096, p=0.001; Model 2, β=-0.116, 95%CI: -0.167,-0.065, p=0.001; Model 3, β=-0.007, 95%CI: -0.118,-0.023, p=0.003). Mediation analysis showed that klotho mediated the statistical association between blood glucose level and cognitive function with proportions (%) of 12.5.

**Conclusion:**

Higher blood glucose levels are associated with poorer cognitive performance in non-diabetic older adults, partially mediated through lower klotho levels.

## 1 Introduction

With global population aging, cognitive impairment has become an important public health issue (1). Numerous epidemiological studies have demonstrated that diabetes mellitus is one of the key risk factors for cognitive decline (2–4). Moreover, increased blood glucose levels are thought to be associated with cognitive impairment even among people without diabetes (5–7). For example, a prospective study found that a reverse U-shaped relationship was observed between fasting glucose and cognitive function, identifying a threshold for highest cognitive performance at 3.97–6.20 mmol/L fasting glucose (5)., indicating impaired fasting glucose may be associated with cognitive impairment. However, associations between blood glucose and cognition are inconsistent across different populations. A study among older Koreans showed that higher blood glucose was only associated with lower memory but not other cognitive domains (7), suggesting potential ethnic differences. Therefore, the relationship between glucose and cognition merits investigation across different ethnicities in the U.S.

The mechanisms underlying the impact of glucose on cognition remain elusive. Some studies have proposed that abnormalities in insulin signaling may be a key pathway (8, 9). In recent years, klotho, an aging-related protein, has gained considerable attention as a key regulator of the insulin/IGF pathway (10). Klotho increases insulin receptor affinity and enhances insulin sensitivity (11). Moreover, klotho exerts neuroprotective effects (12, 13). Hence, klotho may mediate the detrimental effects of glucose on cognition, but relevant evidence is scarce. Given the controversies over the relationship between blood glucose and cognition across populations and the uncertain mechanisms, research leveraging a representative U.S. cohort to examine the impact of glucose on cognition and the potential intermediary role of klotho is warranted. This will facilitate mechanistic understanding of the importance of glucose control for cognitive health.

## 2 Methods

### 2.1 Study population

The data analyzed in this study were from the National Health and Nutrition Examination Survey(NHANES, https://www.cdc.gov/nchs/nhanes/about_nhanes.htm). population. NHANES collects health and nutrition related data through interviews and physical examinations of a representative sample of the civilian non-institutionalized population. Written informed consent was obtained from each participant before participation in this study.

**Figure 1:**
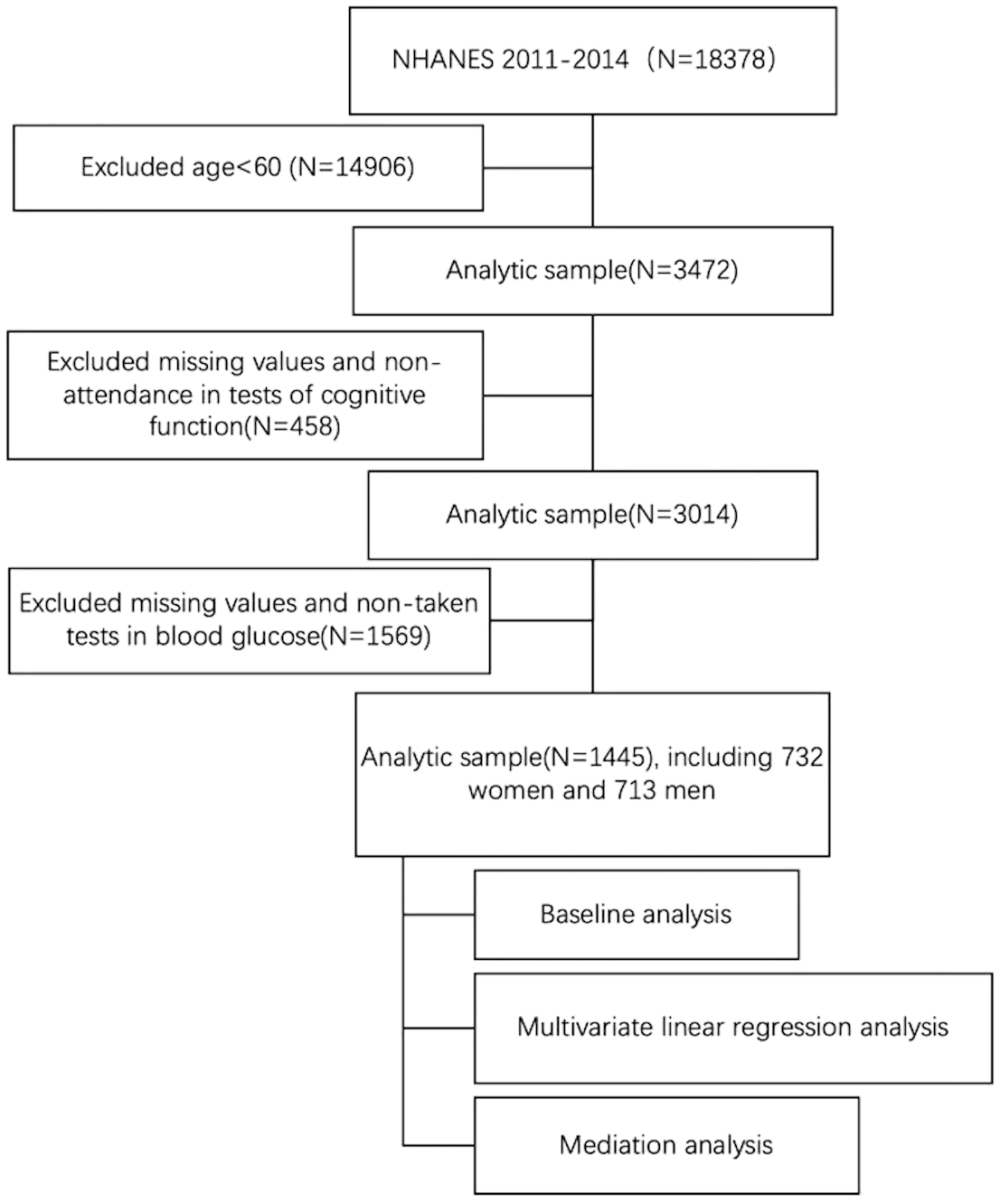
Flowchart of the study design and participants excluded from the study.

Cognitive function of the older population was measured in two cycles of NHANES, 2011-2012 and 2013-2014. A total of 3472 participants were included in the analysis after excluding participants under 60 years of old (n = 14,906). Subsequently, eligible participants needed to have complete data on cognitive function and blood glucose. 458 and 1569 participants who did not participate in the cognitive function test and did not participate in the blood glucose test were excluded, respectively. This resulted in an analytic sample of 1445 participants (see **Figure 1**).

### 2.2 Cognitive function

NHANES performed the Digit Symbol Substitution Test (DSST) for cognitive performance among participants aged 60 years or older. Completing the Digit Symbol Substitution Test (DSST) requires the integrity of executive function, processing speed, attention, spatial perception, and visual scanning cognitive abilities. The exercise is conducted using a paper form that has a key at the top containing 9 numbers paired with symbols. Participants have 2 minutes to copy the corresponding symbols in the 133 boxes that adjoin the numbers. The score is the total number of correct matches. A sample practice test is administered before the participants begin the main test. In NHANES, participants who could not correctly match the symbols with the numbers on their own during the pretest practice did not continue. Details on scoring can be found in the 1999-2000 NHANES CFQ questionnaire data file documentation https://wwwn.cdc.gov/Nchs/Nhanes/1999-2000/CFQ.htm.

### 2.3 Measurement of serum soluble klotho

Prior to analysis, all samples were stored at -80°C. Quantification of Klotho concentrations was performed using a commercially available enzyme-linked immunosorbent assay (ELISA) kit manufactured by IBL International (Japan). The laboratory methodology employed as well as quality assurance and quality control procedures have been described previously in the NHANES study documentation.

### 2.4 Measurement of blood glucose

After a 9-hour overnight fast, fasting blood glucose levels were measured in participants the following morning. These examinations were performed in adherence to established protocols for assessing fasting blood glucose.

### 2.5 Covariates

Covariates evaluated in this analysis included sex (male, female), race/ethnicity (Mexican American, Non-Hispanic Black, Non-Hispanic White, Other), educational attainment (less than high school, high school graduate, college graduate or above), socioeconomic status assessed by Poverty Income Ratio (cite SES source), smoking status (never, former, current), body mass index (<25.0, 25.0-29.9, ≥30.0 kg/m2), and drinking status (never, former, mild, moderate, heavy drinking) based on established categorizations.

### 2.6 Statistical analysis

To obtain population-representative statistics, participants were stratified into quartiles based on the median score of the Digit Symbol Substitution Test (DSST). Categorical variables were summarized as frequencies and percentages, with p-values from chi-squared tests reported. Continuous variables were expressed as mean ± standard deviation (SD), with p-values from Student’s t-tests presented. Associations of DSST scores and Klotho levels with blood glucose concentrations were explored through multivariate linear regression models.

Direct and indirect effects were evaluated to ascertain the mediating influence of Klotho levels on the relationship between blood glucose and DSST performance. Bootstrapping methodology was employed to determine the statistical significance of the mediation pathway. The magnitude of the mediation effect was quantified as the mediation effect percentage, calculated as (mediation effect/total effect) x 100. Statistical analyses were conducted using R statistical software (version 4.2.2, released 2022-10-31, http://www.r-project.org).

## 3 Results

Weighted characteristics of the 1445 participants included in the analyses are shown in **Table 1**. The weighted cognitive function stratified by sex, race, socioeconomic status, education, smoking status, alcohol drinking status, and glucose was statistically significantly different (P < 0.05).

**Table 1:** Weighted characteristics of the study population by Cognitive function.

Variable	level	Quartile 1	Quartile 2	Quartile 3	Quartile 4	*P*-value
n		363	367	364	351	
sex (%)						
	Female	173 (47.66)	150 (40.87)	188 (51.65)	221 (62.96)	<0.0001
	Male	190 (52.34)	217 (59.13)	176 (48.35)	130 (37.04)	
Race/ethnicity (%)						
	Mexican American	50 (13.77)	38 (10.35)	22 (6.04)	16 (4.56)	<0.0001
	Non-Hispanic Black	108 (29.75)	78 (21.25)	75 (20.60)	38 (10.83)	
	Non-Hispanic White	112 (30.85)	179 (48.77)	209 (57.42)	222 (63.25)	
	Other race/ethnicity	93 (25.62)	72 (19.62)	58 (15.93)	75 (21.37)	
Poverty (%)						
	<1	111 (34.47)	56 (16.62)	37 (10.95)	22 (6.79)	<0.0001
	[1, 3)	160 (49.69)	178 (52.82)	145 (42.90)	94 (29.01)	
	≥3	51 (15.84)	103 (30.56)	156 (46.15)	208 (64.20)	
Education (%)						
	low high school	206 (54.21)	192 (55.65)	171 (47.50)	151 (42.18)	0.0017
	High school	94 (24.74)	71 (20.58)	92 (25.56)	90 (25.14)	
	College or above	80 (21.05)	82 (23.77)	97 (26.94)	117 (32.68)	
Smokers						
	former smoker	126 (34.81)	158 (43.05)	148 (40.66)	124 (35.43)	0.0022
	Never smoker	184 (50.83)	157 (42.78)	173 (47.53)	199 (56.86)	
	Current smoker	52 (14.36)	52 (14.17)	43 (11.81)	27 (7.71)	
Alcohol drinkers						
	Former drinker	140 (40.58)	97 (27.02)	91 (25.14)	59 (17.00)	<0.0001
	Heavy drinker	21 (6.09)	42 (11.70)	15 (4.14)	23 (6.63)	
	Mild drinker	83 (24.06)	129 (35.93)	160 (44.20)	184 (53.03)	
	Moderate drinker	22 (6.38)	34 (9.47)	34 (9.39)	44 (12.68)	
	Never drinker	79 (22.90)	57 (15.88)	62 (17.13)	37 (10.66)	
BMI (kg/m2) (%)						
	<25	96 (27.12)	102 (27.87)	93 (25.76)	103 (29.51)	0.8627
	[25, 30)	125 (35.31)	133 (36.34)	123 (34.07)	115 (32.95)	
	≥30	133 (37.57)	131 (35.79)	145 (40.17)	131 (37.54)	
blood sugar (mmol/L) (mean (SD))		6.85(2.57)	6.48(1.93)	6.24(1.59)	6.08(1.54)	<0.0001
klotho (pg/mL) (mean (SD))		818.172 (271.577)	851.201 (304.541)	868.537 (347.222)	864.512 (240.838)	0.2059

Mean ± SE for continuous variables: P-value was calculated by the weighted T test.

% (SE) for categorical variables: P-value was calculated by the weighted chi-square test.

Three multivariate linear regression models were constructed to explore the relationship between glucose and cognitive function (**Table 2**). In the crude model, the standardization coefficient of high glucose and cognitive function is -0.149(95%CI(-0.202,-0.096), p=0.001). After adjusting for sex and race (model 1), the standardization coefficient of high glucose and cognitive function is -0.116(95%CI(-0.167,-0.065), p=0.001). After further adjustment of education attainment, poverty income ratio, smoking status, and alcohol drinking status (model 2), the standardization coefficient of high glucose and cognitive function is -0.070(95%CI(-0.118,-0.023), p=0.003).

**Table 2:** The associations between glucose and Cognitive function.

	Crude model^a^		Model 1^b^		Model 2^c^	
	Standardization coefficientβ(95% CI)	P-value	Standardization coefficientβ(95% CI)	P-value	Standardization coefficientβ(95% CI)	P-value
Glucose (mmol/L)						
<= 6.1	Reference		Reference		Reference	
(6.1,7]	-0.082(-0.135,-0.029)	0.002	-0.069(-0.120,-0.018)	0.008	-0.028(-0.075,0.018)	0.236
>= 7	-0.149(-0.202,-0.096)	0.001	-0.116(-0.167,-0.065)	0.001	-0.070(-0.118,-0.023)	0.003

CI, confidence intervals.

a Crude model: no covariates were adjusted.

b Model 1: sex and race/ethnicity were adjusted.

c Model 2: sex, race/ethnicity, education attainment, poverty income ratio, smoking status, and alcohol drinking status were adjusted.

We further investigated the association between klotho and cognitive function (**Table 3**). After adjusting for all covariates, the results showed that the standardization coefficient between the highest quartile of klotho and cognitive function was 0.091(95%CI 0.031,0.152; p=0.003).

**Table 3:** The associations between klotho and Cognitive function.

	Standardization coefficient β	95% CI	*P*-value
Klotho (pg/mL)			
Q1	Reference		
Q2	0.067	(0.006,0.127)	0.030
Q3	0.095	(0.034,0.155)	0.002
Q4	0.091	(0.031.0.152)	0.003

Adjusted for sex, race/ethnicity , education attainment, poverty income ratio, smoking status, and alcohol drinking statuss.

CI, confidence intervals.

**Figure 2:**
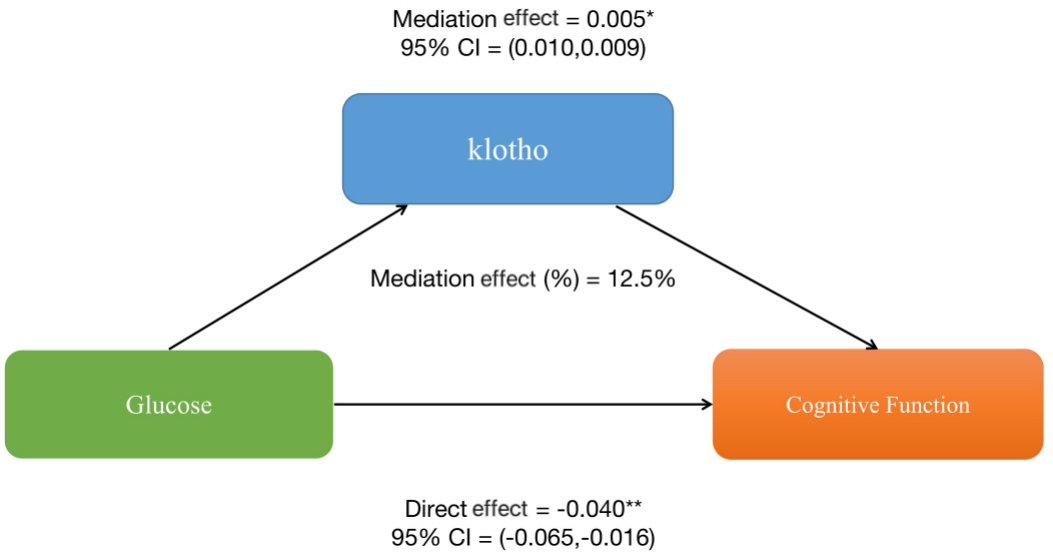
The mediation analysis of klotho on the relationship between glucose and cognitive function.

Furthermore, mediation analyses were conducted to explore the mediating effect of klotho. **Figure 2** shows the mediating role of klotho in the relationship between glucose and sleep cognitive function. Klotho explained 12.5% of the association(p < 0.001).

## 4 Discussion

### 4.1 Main findings

In this nationally representative U.S. sample, we validated the association between higher blood glucose levels and poorer cognitive performance measured by DSST even within non-diabetic range. The linear association was independent of potential confounders including socio-demographics, health behaviors, and adiposity. Our findings corroborate results from several previous epidemiological studies showing dose-response relationships between fasting or postprandial blood glucose levels and cognitive deficits in non-diabetic elderly adults (5–7). The persistent adverse impact of elevated glucose on cognition across the spectrum highlights the need for early screening and preventive interventions even before the diagnosis of diabetes.

Importantly, our study provides novel clinical evidence for the intermediary role of klotho in linking chronic hyperglycemia to cognitive decline. We found that higher serum klotho concentrations were independently associated with better DSST scores. Klotho partially mediated the association between elevated glucose and lower DSST performance. To our knowledge, this is the first population-based study discussing the involvement of klotho in glucose-associated cognitive impairment. The findings align with emerging preclinical evidence indicating neuroprotective properties of klotho against neuronal insulin resistance and oxidative stress (13–15). Our study extends the current literature by identifying klotho as a key intermediary linking circulatory glucose disturbance to cognitive aging in a general elderly population.

### 4.2 Potential mechanisms and clinical implications

#### 4.2.1 Hyperglycemia-induced neuronal damage

Hyperglycemia can inflict neuronal damage through multiple molecular cascades relevant to cognitive decline. Elevated extracellular glucose enhances the formation of advanced glycation end-products (AGEs), which elicit inflammatory responses and oxidative stress in neurons (16). Excessive reactive oxygen species (ROS) production surpasses endogenous antioxidant capacity, resulting in oxidative damage to lipids, proteins and nucleic acids (17). ROS overproduction also activates stress-related signaling molecules including p38 mitogen-activated protein kinase (p38 MAPK) and c-Jun N-terminal kinase (JNK) (18). The activation of p38 MAPK/JNK pathways leads to aberrant hyperphosphorylation of tau, a hallmark of Alzheimer’s disease (19).

Additionally, hyperglycemia disrupts cellular proteostasis by inducing endoplasmic reticulum (ER) stress and mitochondrial dysfunction (20–22). ER stress can elicit neuronal apoptosis through caspase activation. Mitochondrial dysfunction caused by hyperglycemia not only reduces ATP production but also exacerbates ROS generation, establishing a vicious cycle to aggravate oxidative damage in neurons (23). Hyperglycemia also downregulates insulin signaling in the brain by attenuating insulin receptor substrates and Akt signaling, leading to impairments in glucose metabolism and plasticity (24).

These molecular mechanisms represent potential therapeutic targets. Pharmacological agents alleviating oxidative stress, neuroinflammation, ER stress, mitochondrial dysfunction, and insulin resistance could help mitigate hyperglycemia-induced neuronal injury. Lifestyle interventions including exercise, cognitive training, and caloric restriction may also counteract these pathological processes underlying glucose-associated cognitive decline (25–27).

#### 4.2.2 Neuroprotective mechanisms of klotho

**Figure 3:**
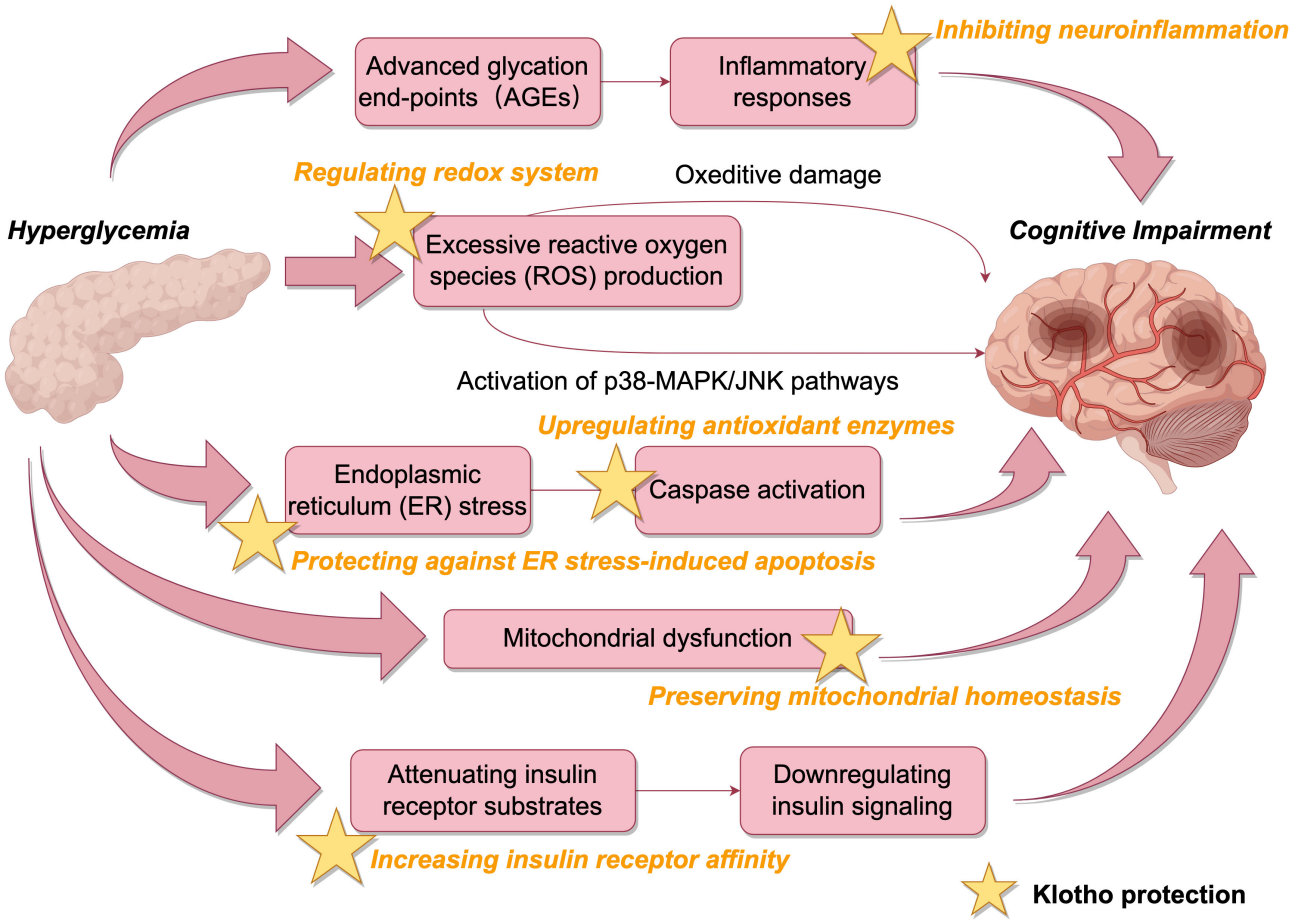
Neuroprotective mechanisms of klotho (By Figdraw).

Klotho may counteract the detrimental effects of hyperglycemia on cognition through diverse mechanisms (**Figure 3**). By increasing insulin receptor affinity, klotho can enhance insulin sensitivity and restore neuronal insulin signaling (28, 29). Through regulating redox systems, klotho suppresses ROS generation and inhibits ROS-induced activation of p38 MAPK/JNK pathways, curbing aberrant phosphorylation of tau (11). Previous studies have shown Sirtuin 1 is involved in insulin release and the regulation of klotho. Klotho may antagonize oxidative stress, activate cellular autophagy, and inhibit neuroinflammation to reduce cognitive impairment by affecting the AMPK/SIRT1 signaling pathway, which plays an important role in glucose regulation, neuron proliferation, oxidative stress, and cognition (30–32).. Klotho also upregulates antioxidant enzymes like superoxide dismutase to bolster neuronal defenses against oxidative damage (33).

Moreover, klotho protects against ER stress-induced apoptosis by blocking ASK1 signaling and inducing autophagy (11). Autophagy degrades misfolded proteins accumulated during ER stress to maintain proteostasis. Klotho preserves mitochondrial homeostasis by inhibiting stress-related signaling molecules and apoptosis pathways (34–36). By alleviating hyperglycemia-induced oxidative, ER and mitochondrial stress, klotho maintains neuronal integrity and function.

Our clinical findings complement these experimental studies to support the neuroprotective role of klotho against glucose toxicity. Further research is needed to elucidate the precise molecular events and signaling pathways linking klotho to neuronal resilience. Uncovering these mechanisms may unveil new possibilities for klotho-based therapies.

Strategies to enhance klotho activity, such as caloric restriction, could offer innovative avenues for mitigating cognitive aging related to glucose dysregulation (37). Exercise training increases klotho levels and improves cognition in older adults, likely through a klotho-dependent pathway (38). Some phytochemicals like curcumin and resveratrol have demonstrated ability to upregulate klotho expression (39, 40). Developing nutritional or pharmacological approaches to boost klotho function warrants investigation for dementia prevention and treatment.

### 4.3 Research strengths, limitations and future directions

Major strengths of the study include the population-based national sample, exploration of a novel intermediary mechanism, and rigorous statistical approach adjusting for potential confounders. However, several limitations should be acknowledged. The cross-sectional nature precludes causal inference. While we selected instrumental variables meeting stringent criteria, residual confounding cannot be excluded. Generalizability to other populations requires further verification. Owing to data constraints, we did not have comprehensive cognitive assessments or neuroimaging biomarkers to enable detailed investigation across cognitive domains.

Future large-scale longitudinal studies incorporating multi-domain cognitive test batteries, neuroimaging, and biomarker assessments are warranted to validate the interrelationships between glucose, klotho, and domain-specific cognitive trajectories. Clinical trials are needed to establish causal impacts of interventions modulating klotho on cognition. Animal models could help elucidate precise molecular mechanisms underlying the cognitive protection afforded by klotho. Elucidating the role of klotho in neuronal maintenance and resilience may uncover innovative prevention opportunities against cognitive aging and dementia related to metabolic disturbance.

## 5 Conclusion

In summary, our study provides novel clinical evidence that higher blood glucose levels are associated with poorer cognitive performance in non-diabetic older adults, partially mediated through lower klotho levels. Our findings underscore the importance of early glycemic control and highlight klotho as a potential intermediary linking glucose disturbance to cognitive aging. Further research into the links between glucose, klotho and cognition may open promising translational opportunities for dementia prevention.

## Data availability statement

The original contributions presented in the study are included in the article/supplementary material. Further inquiries can be directed to the corresponding authors.

## Ethics statement

The studies involving humans were approved by National Center for Health Statistics Ethics Review Board. The studies were conducted in accordance with the local legislation and institutional requirements. The participants provided their written informed consent to participate in this study.

## Author contributions

XL: Conceptualization, Investigation, Writing – original draft, Data curation, Software. YL: Data curation, Software, Writing – original draft, Formal Analysis, Validation. XC: Formal Analysis, Writing – original draft, Conceptualization, Investigation, Methodology. HY: Resources, Visualization, Writing – review & editing. FL: Data curation, Formal Analysis, Investigation, Software, Writing – review & editing. NC: Investigation, Methodology, Resources, Supervision, Writing – review & editing. JC: Project administration, Resources, Supervision, Validation, Writing – review & editing. WL: Project administration, Supervision, Validation, Writing – review & editing.
